# Retraction: Correlation analysis between clinical effective emotional treatment and plasma N-methyl-D-aspartate receptor function-related indexes

**DOI:** 10.3389/fnins.2026.1798060

**Published:** 2026-02-05

**Authors:** Frontiers Editorial Office

The journal retracts the June 2 2025 article cited above.

Following publication, the publisher uncovered evidence that several false identities were used in the peer-review process. The assignment of false reviewers was confirmed by an investigation conducted in accordance with Frontiers' policies and the Committee on Publication Ethics (COPE) guidelines.

Please note that we have not confirmed whether all or any of the authors were aware of or involved in any form of misconduct or manipulation of the publication process.

This retraction was approved by the Chief Executive Editor of Frontiers. The authors received a communication regarding the retraction and had a chance to respond. This communication has been recorded by the publisher.

